# Contemporary Variables that Impact Sleep and Development in Female Adolescent Swimmers and Gymnasts

**DOI:** 10.1186/s40798-021-00331-9

**Published:** 2021-08-09

**Authors:** Janine Bartholomew, Carrie Gilligan, Ann Spence

**Affiliations:** 1 Department of Biology, Portage Learning, 2521 Darlington Road, Beaver Falls, PA 15010 USA; 2 Carlow University, 3333 Fifth Ave, Pittsburgh, PA 15237 USA; 3 Department of Nursing, Carlow University, 3333 Fifth Ave, Pittsburgh, PA 15237 USA

**Keywords:** Gymnasts, Swimmers, Screens, Sleep hygiene, Adolescent female, Social media

## Abstract

The effects of sleep on elite athletes in late adolescence and early adulthood have been well documented in a myriad of sports. However, there is underrepresentation of pre-pubertal and young female adolescent athlete research between the ages of 11–17, and specifically female gymnast and swimmers. Neglecting to understand how high energy demand at a young age relates to sleep and restoration may lead to developmental ramifications for this group, as they display physiological dysfunctions like delayed puberty, amenorrhea and are at risk for the female athlete triad or components of the triad. This review aims to summarize the contemporary variables of blue light emitting screens, social media, and caffeine on quality and quantity of sleep in young athletes while identifying gaps in the literature on how these factors impact the target group of young female swimmers and gymnasts.  The implications of this work include sleep hygiene recommendations for increasing duration and quality of sleep, as well as future research with respect to electronic device usage, social media participation, caffeine consumption, and sport engagement in female early adolescent athletes.

## Key Points

- The sleep habits of young adolescents are changing due to cultural shifts including increased usage of blue light emitting screens, social media, caffeine intake, earlier sports specialization, and demanding schedules.
- While immediate negative effects are linked to decreased sleep quality and time, the long-term consequences in young female athletes have not been explored, especially in those sports linked to the female athlete triad, or components of the triad, and delayed puberty.
- Consideration by coaches, trainers, healthcare professionals, and parents should be given to improving sleep hygiene by focusing on screen usage, caffeine ingestion and social media involvement.

## Introduction

Adolescents are falling short of the American Academy of Sleep Medicine’s (AASM) consensus for sleep (ages 6–12 years require 9–12 h of sleep and 13–18 years require 8–10 h) [1–3]. Cultural shifts in technology usage (i.e., screens and their content) and caffeine consumption, implicate these factors as influencing sleep in adolescence, which is defined as the ages of 11–21 by the American Academy of Pediatrics (AAP) [4]. Sawyer et al. propose a broader definition of adolescence to include the ages of 10–24 years [5]. A secondary analysis of the 2011 National Sleep Foundation (NSF) Sleep in America Poll found adolescents self-reporting sleeping an hour less than the recommended amount, with data indicating almost all reporting using some form of technology an hour before sleep and approximately a third had their cell phone waking them during the night [6]. Additionally, while the AAP does not recommend the stimulant caffeine to be consumed by adolescents [7], 30–50% report ingesting it [8], which may alter their sleep [9]. Youth athletes face these challenges to their sleep with the additional factors of (a) practice, competition, and travel times that may disrupt sleep [10, 11], (b) physical and mental stressors of athletic performance [12], and (c) pain and recuperation due to injury [13].

The International Olympic Committee (IOC) acknowledges sleep as a variable contributing to physiological and cognitive difficulties for young athletes [14].  Indeed, less sleep time was correlated to a decrease in the self-reported well-being of female youth soccer athletes in intensive training [15], while the physical risk of injury increased with less than 8 h of sleep in a survey sample of over 100 athletes [16]. Sleep debt is defined by the Division of Sleep Medicine at Harvard Medical School as “an individual’s accumulated sleep loss from insufficient sleep, regardless of cause” [17]. Sleep duration is used synonymously with total sleep time (TST) and is defined by Kline as “total amount of sleep obtained, either during the nocturnal sleep episode or across the 24-h period” [18]. In adolescents 11 to 15 years old, research by Leger et al. sought to obtain normative data on sleep debt and TST. The authors found that adolescents are getting less TST, and female sleep debt was higher [19].

**Fig. 1:**
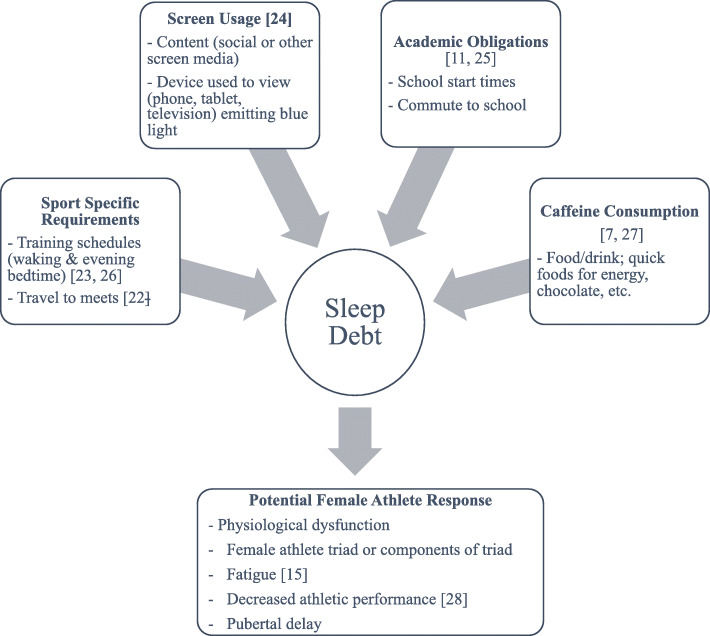
Contemporary independent variable impacting sleep and the potential dependent physiological variables [7, 11, 15, 22–28]. Sleep is broadly indicated in this diagram to include the complex physiological mechanisms that occur as a result of sleep debt. Research by Lo et al. in Hong Kong investigating volleyball, handball, football, and basketball in adolescent athletes found variables such as academics, caffeine consumption, and light were factors in poor sleep quality [11]

Inadequate sleep may contribute to the female athlete triad, which is defined by decreased energy, irregular/delayed menses and decreased bone density [20, 21], in gymnasts and swimmers (Fig. 1).  This research excludes elite pre-pubertal athletes, who present with pubertal delay, affecting skeletal growth and bone acquisition, and amenorrhea [29]. Puberty can be defined by complex hormonal changes and the emergence of secondary sex characteristics, such as those defined as “pre-adolescent” stage 1 [30] (or in some literature, pre-pubertal) to progression to mature stage 5 for breast and genital hair development. There is a void in the epidemiological data for this neglected pre-pubertal stage that may prove to be an important sleep hygiene intervention timeframe, as sleep may influence puberty [31, 32]. This review examines contemporary variables leading to sleep debt in adolescent female athletes (FA) including blue light from screens, social media content and caffeine consumption. This review defines female adolescence as the age range that encompasses the complex pre-pubertal to pubertal stages of development. Given the gap in the literature for adolescent female elite athletes, variables impacting sleep, and altered physiology of female swimmers and gymnasts at risk for pubertal delay in the short term and the triad in the long-term, the importance of identifying recommendations and future areas of research to improve the athlete’s sleep hygiene and overall health is paramount.

## Contemporary Variables in Pre-Pubertal Athletes and the Physiological Implications

The restorative theory of sleep considers physiological mechanisms that rely on the sleep cycles [33], including cell division, growth hormone (GH) release, and energy conservation (or inhibition of depletion of ATP) [33–35]. The theory alludes to physiological implications for sleep as a backdrop for deficient FA, whose physical energy expenditure stretches the limits of physical and mental ability for this developmental stage.   Chronic sleep deprivation may occur in dedicated pre-menorrheal athletes as a result of sport associated reasons like longer or multiple season schedules, travel demands for meets, or training [10, 22, 35], and from contemporary variables (Fig. 1).

**Fig. 2:**
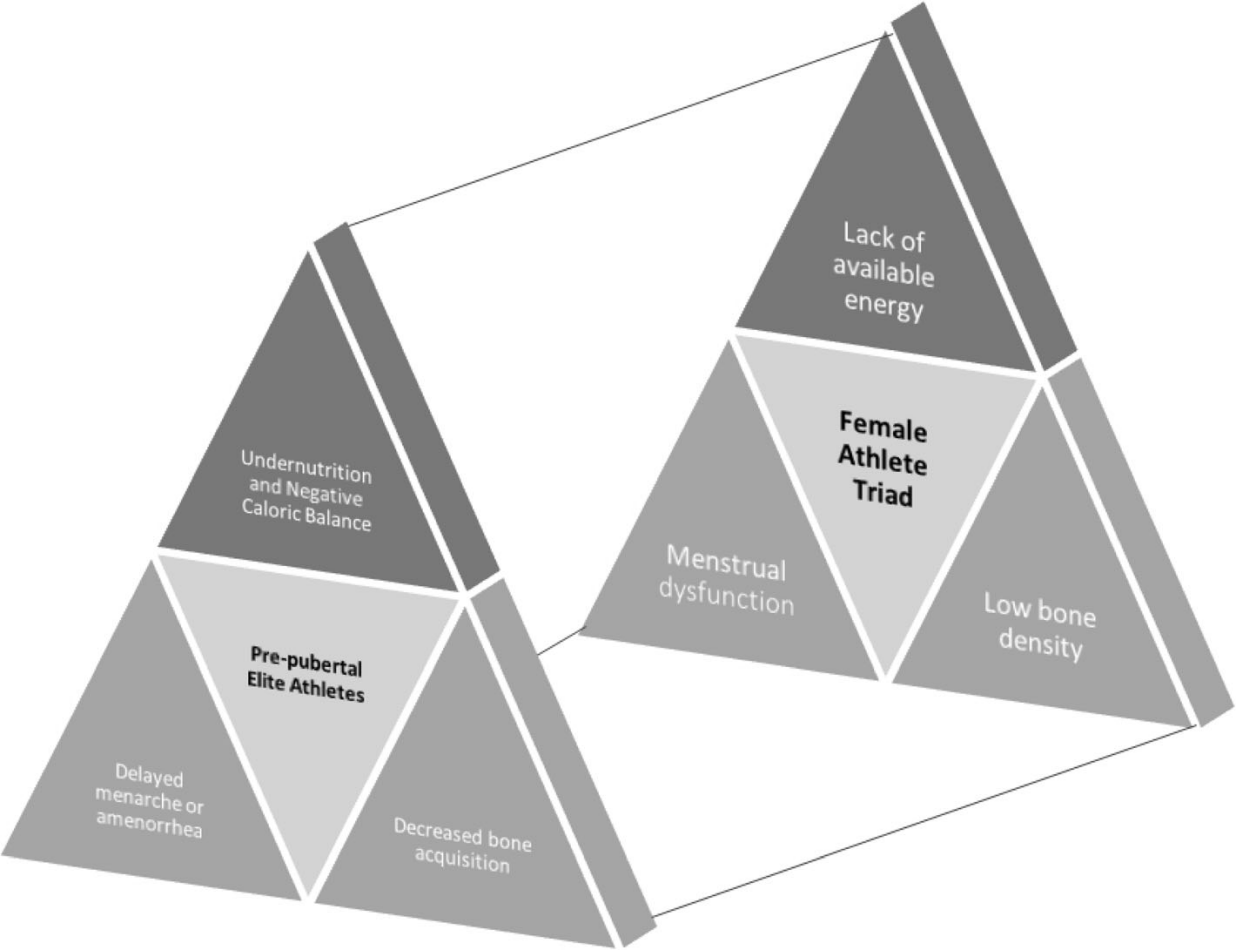
Parallels between altered physiology in pre-pubertal elite athletes and the female athlete triad. The female athlete triad [20, 21, 36] is extensively described in adults with recommendations for adolescent female athletes [37] Evidence is pointing to similar variables and physical demands affecting pre-pubertal athletes, at an earlier stage of development and maturation, and the short- and long-term effects require further empirical investigation

The female athlete triad is observed in post-pubertal athletes, and as Fig. 2 illustrates, its defining features mirror those found in pre-pubertal athletes. While the triad has been understood to be linked to undernutrition [20], sleep [38] may be an underlying factor to components of the triad, for example, low energy availability (LEA) associated with inadequate nutrition. Silva et al. found that 80.7% of gymnasts had an abnormal Epworth Sleepiness Scale (ESS) and 77.6% had an abnormal Pittsburgh Sleep Quality Index (PSQI) [39]. Recent work from Silva et al. indicates that 91.5% of gymnasts (12.8 ± 3.1 years old) slept less than 8 h per night, and 60% had observable features of the triad with amenorrhea and decreased energy intake and availability [40]. This decrease in sleep can influence hormonal release and appetite among other homeostatic body mechanisms which, as cited by the authors, impacts “…mood, performance and recovery…” of body functions [40]. In a study by Silva and Paiva athletes with menstrual irregularities in intensive engagement in gymnastics displayed decreased energy availability, body fat, and insufficient nutrition, i.e., low calcium intake among other nutrients, which may lead to issues in developing bone [41].  Similar to their gymnast counterparts, synchronized swimmers with average age of 20.4, demonstrated decreased sleep at 5.7 h per day and decreased intake of calcium, iron, and fiber as indicated by Costa et al. [42]. Interestingly, an increased sleep time was associated with decreased menstrual irregularities in Korean female adolescents after the authors controlled for confounding variables, such as menarcheal age and BMI among others [43]. Additionally, intensive training load as indicated by Dumortier et al. is a variable that negatively impacts both TST and athletic performance in adolescent female elite gymnasts [44]. As Gudmundsdottir reports, TST is also a variable for swimmers with early morning training sessions, finding that TST is decreased the night before early morning tapering and the shortest sleep duration followed days of no training [23]. Taken together, this review posits that sleep should be considered in the altered physiology observed in pre-pubertal adolescent female athletes in gymnastics and swimming as it parallels the features of the triad (Fig. 2).

### Activity-Induced Menstrual Dysfunction or Pubertal/Menarche Delay

Activity-induced amenorrhea or delayed puberty onset is associated with a negative energy balance and low body weight and is observed in athletes like gymnasts [45]. Schtscherbyna et al. found that in 78 elite female swimmers aged 11–19 years old, one component of the triad was present in 47% while two components were present in 15.4% and 1.3% for all components [46]. Female swimmers may exhibit low energy that may impact physiological functions such as normal menstruation [47] in the same way as their gymnast counterparts. In a sample of four competitive swimmers, ages 15–19, with a control group of highly active females aged 16–18 with comparison data from normally menstruating women, Bonen et al. found that compared to controls, there was a significant reduction in the FSH to LH ratio over the entire menstrual cycle, among other findings [48]. The authors observed anovulation in their sample of swimmers [48]. Further, some samples of swimming FA have presented with oligomenorrhea [49].

A study by Hoch et al. that compared 80 high school varsity athletes to 80 sedentary high school students found 54% of the athlete group had menstrual dysfunction versus 21% of sedentary students [50]. Both groups had normal TSH, prolactin, and no difference in FSH, luteinizing hormone (LH), or estradiol [50]. The consequences of high intensity athletic training in the pre-pubertal athlete and its long-term consequences are less documented. The energy demand may outpace the body’s means of energy restoration in this group. It should be noted that the American Academy of Pediatrics highlights that training hours not exceed an athlete’s age in years per week but recognizes that longitudinal evidence is needed [51]. However, Root et al. recorded that over half of the gymnasts > 11 years old exceeded the recommendation [52]. Delays in the pubertal growth spurt, first menarche and skeletal maturation were observed in young FA, training at the minimum of 15 h/week [53]. The restorative sleep theory would posit that sleep grants a time for an inhibition of ATP depletion, and energy would be conserved. However, this group may also be at risk for sleep debt at a time when their physiology would naturally start increasing sleep. Laberge et al. observed a maturation associated increase in sleep starting at 10–13 years old, with sex related differences in the amount of sleep (based on time in bed), with females sleeping more than their male counterparts, as the timing of maturation is earlier in females [54].

Shaw et al. studied puberty development and the relationship between sleep and pulsatile LH secretion, which is needed for the onset of puberty [31]. Results of this study indicate that sleep, specifically slow wave sleep (SWS), contributes significantly to the rise of LH secretion needed for development [31]. Work in adult marathoners, demonstrating increased energy demand from high exertion, indicates that a part of the recuperation included proportional increases in SWS [55]. However, specific evidence showing whether sleep restriction in pre-pubertal or adolescent athletes affects time spent in SWS is lacking. This may be consequential because Shaw et al. hypothesize that deep sleep or SWS is necessary because deep sleep occurs 5–15 min before the LH pulse [31]. Research by Taylor et al. suggests that in a sample of seven female swimmers with a mean age of 19 years, SWS as a percentage of total sleep time increases at the height of the season and reduced significantly at the taper phase of the season [56].

One of the variables impacting sleep in pre-pubertal athletes is caffeine. It should be noted that there is a gap in the literature exploring the use of caffeine in pre-pubertal females and its potential effects on the endocrine system. While human studies are lacking, animal studies show that caffeine may be a potential endocrine disruptor by interfering with the hypophyseal-pituitary-gonadal axis [57].

### Bone Acquisition and Bone Mineral Density

Sleep is linked to anabolic pathways [34] so how sleep disturbance affects metabolic pathways in young athletes needs to be considered. Dattilo et al. hypothesize a shift towards catabolic hormones during sleep debt contributes to muscle mass loss and a decrease in muscle recovery after exercise or injury [58].  Relevant to the pre-pubertal female athlete are malnutrition [29] and sleep deprivation which affect the anabolic growth hormone (GH) [59]. GH and insulin-like factor-1 (IGF-1) regulate the linear growth in children [29], and intensive training in pre-pubertal athletes prior to the pubertal growth spurt may alter GH and IGF-1 secretion, inhibiting linear growth. These athletes shift their maturation to a later age, demonstrated by attaining their projected height after sport retirement or decreased training [29]. However, the physiological implications of delayed skeletal system maturation has not been elucidated.

The highest rate of bone accumulation in females is during puberty [60]. One of the primary concerns of amenorrhea or delayed puberty is a hypoestrogenic state and its short and long-term effects on bone acquisition and bone mineral density (BMD). Lerand and Williams indicate that during the window of bone growth when young women gain the typical 2–4% of bone mass, the occurrence of amenorrhea or oligomenorrhea can result in a loss of 2% annually [36]. Data from female gymnasts (aged 13–23 years) indicate the time of onset of strenuous exercise has a negative impact on bone acquisition [61], and delayed puberty is associated with increase in fracture risk during adolescence [62]. Women (not associated with athletic participation) with late menarche correlated with having lower bone mineral density and observational studies show age of menarche may be an indicator of risk of osteoporosis in pre- and postmenopausal years [62]. As well, approximately a third of former collegiate gymnasts reported disordered eating and a significant 4% of these athletes had osteoporosis [63]. It should be noted that a study examining bone mineral density, body composition, dietary intake, and energy use in pre-menarcheal gymnasts aged 10–15 indicated that their bone mineral density was higher than matched controls [64] as many suggest that it is an osteogenic sport. As indicated by Siatras and Mameletzi, the weight bearing benefits of the sport of gymnastics and the resultant increased bone mineral density on specific areas of bone may not be enough to counter the impact of “hormonal deficiency” that is a result of sustained energy debt [65]. Evidence from Duchar et al. suggests that retired gymnasts may lose some of the osteogenic advantages of the sport due to a health history of amenorrhea versus their gymnast peers who did not have a history of menstrual irregularities [66]. However, swimming is a decreased gravity sport and is not considered osteogenic [67–69]. In elite female aquatic athletes, Bellver et al. indicate that BMD is higher in non-aquatic athletes than aquatic athletes, and their research suggests integrating aquatic sport with weight bearing activity [70] consistent with suggestive evidence from other researchers [71]. Research by Gomez-Bruton et al. that included males, demonstrated that female swimmers with a mean age of 13.59 ± 1.94, displayed decreased BMD compared to controls [68]. The female participants only demonstrated higher BMD as compared to controls in the upper extremity [68]. In collegiate female swimmers compared with other sports, such as gymnastics, track, and basketball among others, swimmers display reduced mean leg BMD [72]. Additionally, in a 9-month longitudinal study of male and female adolescent swimmers Ribeiro-dos-Santos et al. found a negative relationship to BMD increase irrespective of sex, which accompanied longer adolescent and child participation in the sport [73].

Caffeine’s influence on calcium balance and resulting bone acquisition/BMD in children is lacking in the literature. However, 30–50% of adolescents report consuming energy drinks which may contain caffeine [74] and in some student athlete samples a higher percentage of energy drink consumption is evident [75]. Questionnaires from students with an average age of 14.3 show that 69% of young athletes consume energy drinks with 17% drinking them daily or 1–3 times per week [75]. According to the American Academy of Pediatrics (AAP), adolescence appears in three stages, early, middle, and late in the age range of 11–21 [4] and caffeine consumption is not recommended by the AAP for adolescents [7]. Experiments in adults indicate an increase in urinary excretion of calcium, magnesium, sodium, and chloride up to 3 h after ingesting caffeine. This level of loss was not detrimental to BMD in healthy young women [76]. However, in postmenopausal women, with inadequate calcium levels, consuming high caffeine is associated with negative changes to BMD and bone loss compared to those consuming less caffeine [77].  Barger-Lux and Heaney report a meta-analysis of 560 studies that showed 6 fl oz (177.5 ml) of coffee negatively affected calcium by 4.6 mg/day, not dependent upon low calcium intakes or populations with decreased estrogen [78].  This information may be relevant to the pre-pubertal athlete, whose training may delay menarche producing a low estrogen state similar to the postmenopausal state. It may be additionally relevant if adolescent FA present with calcium deficiency. Investigations should be done to determine whether studies on caffeine consumption and calcium balance in postmenopausal women provide better comparatives to prepubertal athletes or those with amenorrhea than premenopausal women.

Proper education and intervention may improve long term outcomes, as Barger-Lux and Heaney suggest increasing calcium intake by 40 mg for every 177.5 ml of caffeine containing coffee [78]. The importance of calcium in swimmers [79] and gymnasts [80] has been emphasized in the literature. While there are still numerous unanswered questions related to sleep and bone metabolism in female athletes, research is starting to elucidate the impact of disruptions in circadian rhythm and bone in adults. Bone resorption processes peak at night, and interruption of sleep, in terms of duration and timing, could upset the balance in this physiological process. Swanson et al. discuss the linkage between night shift work and low bone mineral density as well as the impact of caffeine in reducing gastrointestinal calcium absorption [81]. The authors hypothesize altered bone turnover markers due to disrupted sleep can shift the balance of bone resorption/formation and perhaps influence bone mass/quality and the risk of fracture [81]. Research in rats indicates chronic sleep deprivation decreases bone mineral density [82].

## Factors Impacting Adolescent Sleep in the General Population and Youth Athletes

Sleep hygiene includes the best practices to increase the quality and quantity of sleep in an individual and the habits and behaviors to maintain proper sleep health [83]. As a way to investigate the best practices that would improve sleep hygiene in adolescent FA, the authors of the current review sought to find lifestyle habits that may possibly hinder sleep. Below are the findings of a literature search on how social media participation, screen exposure, and caffeine consumption may impact sleep (Fig. 1). General population and studies specific to athletes are discussed.

### Blue-Light Emitting Screens and Social Media Content

Numbers from the Pew Research Center indicate the majority of US youths are exposed to screens, with 88% of American teens reporting having access to a mobile phone, 87% to a computer, and 58% to a tablet [84]. Other sources that are grouped into ‘screens’ because of content and light emitted are televisions, e-readers, and watches connected to smartphones. The direct influence of mobile devices and social media usage on the sleep hygiene routine is apparent in multiple studies [6, 85, 86] and is a major health concern. Technology usage and screen time may be adding to this deficit. Other studies indicate that media viewed on devices can also impact sleep, as it infringes on the time children have to sleep [87], increases arousal, and stress [88]. Lastly, the engagement in games and social interaction cause the brain to be alert and delays sleep [89]. Burke uniquely points to the matter of the content of the media usage [90]. Should the images in media usage evoke negative emotion, the potential for the athlete to be unable to fall asleep at an assigned time may be disturbed secondary to unease. Van der Schuur et al. found sex differences in social media usage and sleep, specifically, positive correlations between social media stress and sleep latency in girls [91]. Sleep latency is defined as “how long it takes a person to fall asleep from the onset of the potential sleep period” [17].

Recent research by Scott et al. examining 11,872 UK adolescents found a correlation between high social media usage and inadequate sleep patterns. The authors found that females were higher users of social media than males [92]. Research by van der Schuur et al. examined social media use in relation to stress and sleep in adolescents. The study included 1441 adolescents from 11 to 15 years old who filled out a survey in one to three waves [91]. The most significant finding in this study revealed that social media stress rather than the frequency of usage may be associated with decreased sleep, and females were influenced more by the stress [91]. While it is difficult to separate the variables of the content viewed on a screen from the blue-light emitted from the screen, work is being done to elucidate the effects of screens on physiology [88].

Screens have also been shown to interfere with total sleep time and daytime sleepiness because of short wave-length light emission. According to Chang et al., the light that comes from electronic devices can interrupt the circadian rhythm [93]. The use of short wavelength blue light emitted from screens interrupts the circadian clock which can suppress melatonin production needed for sleep. This change in the circadian rhythms makes it more difficult to fall asleep. The bright light keeps the brain alert creating wakefulness and a further inability to fall asleep [89]. Light type, whether bright, red, or blue are also variables influencing sleep and athletic performance. Research by Zeitzer et al. illustrates that even low levels of bright light can influence circadian rhythms [94]. Zhao et al. investigated the positive use of red light with Chinese female basketball players. The authors used red light from a machine that emitted “… an average wavelength of 658 nm and light dose of 30 J/cm2”, finding that the light improved sleep quality according to the Pittsburgh Sleep Quality Index and also increased serum melatonin levels [95].

An adult study investigating shift work and melatonin indicates bright light exposure creates a marked decrease in melatonin compared to conditions of dim light or light filtering goggles [96]. This demonstrates that bright light impacts sleep physiology. Empirical and replication studies are needed to link a specific period of time to sleep after using bright screen light in adolescent females and the physiological consequences of longitudinal exposure. While some studies offer specificity with regard to devices that emit bright screen light and *behavior* with sleep, e.g., mobile device usage and time, the physiological consequences appear to be lacking in the literature to the best of our knowledge for this population. For example, Bartel et al. examined 63 adolescents and phone usage prior to bed and found that those who stopped usage of their device 80 min earlier, gained 21 more minutes of sleep time each night of the study [97].

Such forms of light may be influential to the young female athletes’ sleep start time (SST). Knufinke et al. conducted a study of male and female young athletes, finding that during 70% of the nights in the study, athletes engage in blue light activity before sleep [98]. Both swimmers and gymnasts are exposed to bright light at various times of the day dependent upon meet times, and across summer or winter seasons at different times of the day.

It is not surprising that in studies investigating media usage and sleep habits, there are large amounts of usage with mobile devices. The ease by which the mobile device can be accessed and used for multiple purposes is apparent including: (a) texting, (b) social media, (c) gaming, (d) video exchange, and (e) streaming content. Brunborg et al. investigated a sample of over 800 in a wide age range, finding that over 42% of the participants engage in mobile device use in their sleep area everyday [99]. Some studies report higher numbers in their sample [6].

### Caffeine Consumption

The impact of caffeine in today’s youth cannot be ignored. Caffeine is contained in numerous drinks and snacks consumed by youth today including tea, sodas, highly sought-after specialty coffee drinks, and chocolate [100]. Considering accessibility and consumption, adolescents in research conducted by Thakre et al. were not able to accurately identify caffeine content in common and readily available drinks such as carbonated beverages and tea [101]. In an effort to study the effect of caffeine on children, Aepli et al. studied 32 children between the age of 10 and 16.9 years [9]. Data was collected via subject questionnaires, which included reporting caffeine intake, and reporting on morning tiredness by using a visual analog scale. Sleep assessment was obtained through electroencephalogram (EEG) recordings. Results indicate that caffeine consumption is associated with later bedtimes and thus a shorter time in bed. Subjects who consumed caffeine also had reduced sleep depth as measured by the slow wave activity (SWA) [9]. Another study done by Watson et al. sought to examine the relationship between caffeine consumptions and sleep and behavior in children 8–12 years old [102]. Questionnaires were given to both parents and children regarding caffeine intake and quality of sleep. There was a total of 309 participants who were an average age of 10.6 years. In this study, 87% of the children reported consuming caffeine. Watson et al.’s post-hoc analysis identified a difference in caffeine consumption and total sleep time where those who slept 9–11 h consumed less caffeine than those sleeping 7–8 h [102].

Although further studies need to be conducted to determine the exact effect of caffeine on adolescent athletes there is evidence to support that caffeine affects the quality and amount of sleep in children [103]. Research by Drake et al. suggests that caffeine intake as far as six hours from a designated bedtime may impact sleep [104]. There is the potential for athletes to engage in unhealthy drink and food choices that may delay onset of sleep, i.e., chocolate, or carbonated beverages.

## Recommendations

This integrated literature review has established that sleep is important [105] for restoration and that sleep deprivation may lead to diminished adolescent FA wellness in gymnasts and swimmers. Several studies provide explanation for habits that may influence the amount of sleep in adolescents. One study found that on average adolescents self-report sleeping an hour less than recommended, and that they use some form of technology an hour before sleep [6]. The screens have been shown to interfere with total sleep time and daytime sleepiness because of short wave-length light emission. The authors report that the light that comes from electronic devices can interrupt the circadian rhythm, suppress melatonin secretion, and keep the brain alert and unable to fall asleep [89, 93]. Thus, a potential recommendation, in line with several authors is to establish healthy bedtime habits and routines [106] that minimize exposure to blue light emitting devices, such as mobile devices [97]. As evidenced by Perrault et al. a possible restriction of screen usage after 21:00 h to improve total sleep time may be another recommendation [107]. Current research is also elucidating whether the brightness of the screen is a variable; see Nagre et al. [108] and Mouland et al. [109]. Studies looking at sleep also found that caffeine is frequently consumed by adolescents and is correlated with 2–3 h less sleep, suggesting caffeine intake should halt 6 h prior to bed [9, 102, 104]. Bartel et al. indicate that limiting mobile phone use 60 min prior to bedtime [97] may be a possible routine to improve sleep duration. In some research, the condition of 30 min of restrictive mobile use is evident in a sample of females and males in their early twenties [110]. Future recommendations should be based upon physiological data. In young adults, ages 19–32, social media usage 30 min prior to bedtime interferes with sleep [111], thus additional research is needed to investigate the adolescent female athlete population in particular, female swimmers and gymnasts.

The results of these studies suggest that a sleep hygiene routine could be implemented to help mitigate the barriers to optimal sleep for both swimmers and gymnasts. Healthcare professionals, coaches, trainers, and parents are in a unique position to counsel female adolescent athletes about the risk of sleep deprivation and the benefits of a sleep hygiene routine. Healthcare professionals have an opportunity to coordinate care for the adolescent FA in both an interdisciplinary and multidisciplinary context. The current caffeine habits of female adolescent athletes should be investigated in the delayed menarche group already prone to altered BMD as caffeine or its effect on sleep may alter bone physiology. Proper education and intervention may improve long term outcomes, as Barger-Lux and Heaney suggest, “…increasing calcium intake by 40 mg for every 177.5 ml of caffeine containing coffee” [78].

## Conclusions

Considering studies that indicate sport specialization is occurring earlier in adolescent development [112, 113], these authors posit that there will be an increased need to examine the physical demands of specific sports in relationship to female adolescent development and the surrounding variables of diet, sleep, technology habits, roles, and routines (Table 1). Nédélec et al. suggest that mixed methods research regarding athletes and sleep should examine sleep, and the holistic variables that surround sleep [114]. Mixed methods research with adolescent females specific to sports with different physical and time demands may assist health researchers and clinicians alike, who seek to prevent health difficulties specific to adolescent FA still in the phases of pubertal growth and development (Table 1).

**Table 1:** Future research questions

Research question	Possible mixed methods inquiry
Does screen usage and caffeine consumption, prior to sleep impact the adolescent female gymnast and swimmer athlete performance and sleep quality?	PSQI; sleep tracking devices, including the sleep timer function of mobile devices; individual interviews; diet diary; sports season results
Does screen content and caffeine consumption impact the adolescent female gymnast and swimmer sleep quality?	PSQI; Electronic social media, text, video diary or emoji questionnaire; sleep tracking devices; diet diary; individual interviews
Is SWS and subsequent hormone release impacted by routines prior to sleep including screen usage and caffeine consumption in adolescent female gymnasts and swimmers?	Growth hormone testing; LH testing; portable EEG device testing

Different independent and dependent variables are highlighted. Different sports could be substituted in the place of gymnast and swimmer. Screen content and screen usage separated, although the two could become confounding variables since blue light and screen content can occur simultaneously on a screen. Future research would need to define screen usage and the technological variables associated with that usage, i.e., social media, texting, video, and light emitted. The PSQI is a measure of sleep quality [115]. A social media diary may include content viewed during the day and emotional perception of that content. Individual interviews may provide information without peer influence.

Numerous quantitative measures for examining sleep are well evidenced, for example, the Pittsburgh Sleep Quality Index (PSQI) which demonstrates reliability and validity in adolescents [115] and could be used with this adolescent population in mixed research methodology. There are also technological options related to investigating sleep in this population, which may provide quantitative information, non-invasive to a typical sleep hygiene routine. Lower cost wearable sleep tracking devices, such as rings, have provided sleep data in adults correlated to actigraphy [116]. However, where quantitative methods fall short of examining intricacies of the female athlete daily routine variables in specific sports, the gap may be filled by utilizing qualitative methods to understand those intricacies in large groups of female athletes in specific sports.

This narrative review has examined the specific components related to two physically demanding sports: gymnastics and swimming. As both sports require year-round engagement at dedicated competition levels, and high physical demand, it is hypothesized that there is a solid basis for investigating the variables surrounding female adolescent athletes in those individual sports (Fig. 1). Since quality sleep is essential for the human condition, and restorative, the study of sleep in adolescent females could provide preventative information for parents, coaches, physicians, nurses, and therapists working with adolescent FA. Recent data show a propensity toward specific training in one sport, sport specialization, at early ages [117]. As research continues with greater prevalence in the area of athletes and sleep, it is recommended that greater specificity follow in the research stream as related to sport specialization and the unique nature of the adolescent female swimmer and gymnast.

## Data Availability

Not applicable.

## Abbreviations

ATP: Adenosine triphosphate
ESS: Epworth Sleepiness Scale
EEG: Electroencephalogram
FA: Female athletes
GH: Growth hormone
IOC: International Olympic Committee
LH: Luteinizing hormone
NREM: Non-rapid eye movement
PSQI: Pittsburgh Sleep Quality Index
SWS: Slow wave sleep
